# Adverse in‐hospital outcomes in patients with paraplegia who undergo radical prostatectomy

**DOI:** 10.1111/bju.70021

**Published:** 2025-10-10

**Authors:** Andrea Marmiroli, Francesco Di Bello, Natali Rodriguez Peñaranda, Mattia Longoni, Quynh Chi Le, Fabian Falkenbach, Michele Nicolazzini, Calogero Catanzaro, Zhe Tian, Jordan A. Goyal, Stefano Luzzago, Francesco Alessandro Mistretta, Mattia Piccinelli, Fred Saad, Shahrokh F. Shariat, Alberto Briganti, Felix K. H. Chun, Salvatore Micali, Nicola Longo, Markus Graefen, Carlotta Palumbo, Riccardo Schiavina, Gennaro Musi, Pierre I. Karakiewicz

**Affiliations:** ^1^ Cancer Prognostics and Health Outcomes Unit, Division of Urology University of Montréal Health Center Montréal Québec Canada; ^2^ Department of Urology IEO European Institute of Oncology, IRCCS Milan Italy; ^3^ Università degli Studi di Milano Milan Italy; ^4^ Department of Oncology and Haemato‐Oncology Università degli Studi di Milano Milan Italy; ^5^ Vita‐Salute San Raffaele University Milan Italy; ^6^ Division of Experimental Oncology/Unit of Urology, URI, Urological Research Institute IRCCS San Raffaele Scientific Institute Milan Italy; ^7^ Department of Neurosciences, Science of Reproduction and Odontostomatology University of Naples Federico II Naples Italy; ^8^ Department of Urology, Ospedale Policlinico e Nuovo Ospedale Civile S. Agostino Estense Modena University of Modena and Reggio Emilia Modena Italy; ^9^ Division of Urology, Department of Translational Medicine, Maggiore della Carità Hospital University of Eastern Piedmont Novara Italy; ^10^ Division of Urology IRCCS Azienda Ospedaliero‐Universitaria di Bologna Bologna Italy; ^11^ Department of Urology, University Hospital Goethe University Frankfurt Frankfurt am Main Germany; ^12^ Martini‐Klinik Prostate Cancer Center University Hospital Hamburg‐Eppendorf Hamburg Germany; ^13^ Department of Urology, Comprehensive Cancer Center Medical University of Vienna Vienna Austria; ^14^ Department of Urology Weill Cornell Medical College New York NY USA; ^15^ Department of Urology University of Texas Southwestern Medical Center Dallas TX USA; ^16^ Hourani Center for Applied Scientific Research Al‐Ahliyya Amman University Amman Jordan

**Keywords:** prostate, NIS, paraplegia, complications, morbidity

## Abstract

**Objective:**

To test for the association between paraplegia and perioperative complications as well as in‐hospital mortality after radical prostatectomy (RP) for non‐metastatic prostate cancer.

**Patients and Methods:**

We identified patients who underwent RP (National Inpatient Sample [NIS] 2000–2019), stratified according to paraplegia status. The NIS is an inpatient database that rests on data contributed by ~20% of community hospitals within the United States. Descriptive analyses, propensity score matching (PSM, ratio 1:10), and multivariable logistic regression models (LRMs) were used.

**Results:**

Of 260 302 patients who underwent RP, there were 223 (0.1%) with paraplegia. The patients with paraplegia who underwent RP were younger (age 60 vs 62 years; *P* = 0.002) and more frequently had Charlson Comorbidity Index ≥3 (46% vs 2.2%; *P* < 0.001). After 1:10 PSM, 223/223 (100%) patients with paraplegia and 2230/260 079 (0.9%) without paraplegia who underwent RP were included in further analyses. In multivariable LRMs, patients with paraplegia who underwent RP exhibited significantly higher in‐hospital mortality (adjusted odds ratio [aOR] 10.7), higher rates of wound complications (aOR 8.2), infectious complications (aOR 6.2), genitourinary complications (aOR 3.5), intraoperative complications (aOR 2.8), cardiac complications (aOR 2.8), pulmonary complications (aOR 2.6), overall complications (aOR 2.4), blood transfusions (aOR 1.8), and longer length of stay ≥75th percentile (aOR 1.7) (all *P* ≤ 0.01).

**Conclusion:**

Although patients with paraplegia who undergo RP are rare, adverse in‐hospital outcomes are substantially more frequent in these individuals. These observations should be carefully considered in clinical decision making and informed consent prior to RP, if such procedure is contemplated in patients with paraplegia.

**Abbreviations**
CCI: Charlson Comorbidity Index
GEE: generalized estimation equation
ICD(‐9)(‐10)(‐CM): International Statistical Classification of Diseases and Related Health Problems (ninth revision) (10th revision) (Clinical Modification)
LRM: logistic regression model
NIS: National Inpatient Sample
(a)OR: (adjusted) odds ratio
PCa: prostate cancer
PSM: propensity score matching
RP: radical prostatectomy

## Introduction

Radical prostatectomy (RP) is a guideline‐recommended standard treatment for non‐metastatic prostate cancer (PCa) [1, 2]. Occasionally, RP may be indicated in patients with paraplegia, who represent a limited proportion of the overall population with PCa (0.1%) according to existing medical literature [3, 4, 5]. Under such circumstances, paraplegia may predispose to higher rates of complications and in‐hospital mortality after RP [6]. Indeed, paraplegia is associated with higher rates of adverse in‐hospital outcomes after radical cystectomy [7]. Data examining other major cancer surgeries are scarce. This knowledge gap also applies to RP. To address the lack of data regarding adverse in‐hospital outcomes in patients with paraplegia, we tested the association between paraplegia and adverse in‐hospital outcomes within a large‐scale population‐based cohort of patients with non‐metastatic PCa undergoing RP, using data from the National Inpatient Sample (NIS 2000–2019). We hypothesised that patients with paraplegia may exhibit higher adverse in‐hospital outcomes compared to those without paraplegia when RP is performed.

## Patients and Methods

### Data Source

To test for perioperative complications and in‐hospital mortality after RP, we relied on discharge data from the NIS (2000–2019). The NIS is a set of longitudinal hospital inpatient databases included in the Healthcare Cost and Utilization Project (HCUP), formed by the Agency for Healthcare Research and Quality (AHRQ) through a Federal‐State Partnership and based on data contributed by ~20% of community hospitals within the United States [8]. All diagnoses and procedures were coded using the International Statistical Classification of Diseases and Related Health Problems (ICD), ninth revision‐Clinical Modification (ICD‐9‐CM), ICD, 10th revision‐CM (ICD‐10‐CM), as well as ICD‐10‐Procedure Coding System (ICD‐10‐PCS).

### Study Population

**Fig. 1:**
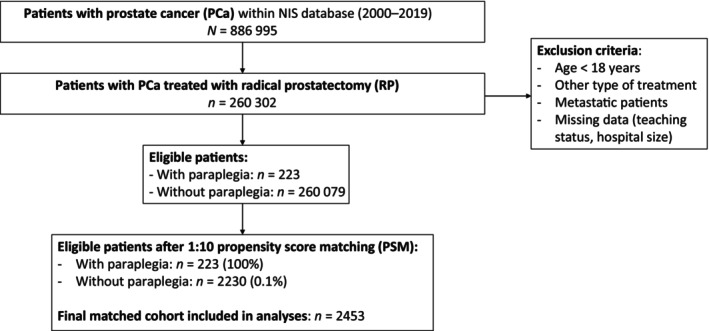
Flow diagram of the patients’ selection.

We focused on patients with a primary diagnosis of non‐metastatic PCa (ICD‐9‐CM codes 185, and ICD‐10‐CM code C61) aged ≥18 years (Fig. 1). Only patients treated with RP were included according to previously reported methodology [9]. Additionally, patients were stratified according to paraplegia status (ICD‐9‐CM codes 344.1, 342.x, 334.1, 342.x, 343.x, 344.0–344.6, 344.9 and ICD‐10‐CM codes G04.1, G11.4, G80.1, G80.2, G81.x, G82.x, G83.0–G83.4, G83.9) [10].

### Definition of Variables for Analyses

Study endpoints comprised adverse in‐hospital outcomes. These consisted of infectious, pulmonary, genitourinary, wound, cardiac, vascular, gastrointestinal complications and blood transfusions, length of stay ≥75th percentile (≥3 days), and of in‐hospital mortality (Table S1). All characteristics were identified using ICD‐9 and ICD‐10 codes according to previously established methodology [11, 12]. Comorbidities were defined according to the Deyo modification of the Charlson Comorbidity Index (CCI) [13]. Covariates consisted of patient characteristics including age at admission (years, continuously coded), CCI (0–1 vs 2 vs ≥3), as well as hospital characteristics including teaching hospital status (teaching vs non‐teaching), and hospital size (large [≥400 beds] vs medium [200–399 beds] vs small [<200 beds]). As the group of interest within the present study exclusively consisted of patients with paraplegia, we examined the effect of paraplegia on adverse in‐hospital outcomes without including this characteristic within the CCI calculation according to previously reported methodology [14].

### Statistical Analyses

First, patient and hospital characteristics as well as perioperative length of stay, perioperative complications and in‐hospital mortality rates were tabulated. Medians and interquartile ranges (IQRs) were calculated for continuously coded variables. Frequencies and proportions were computed for categorical variables. Wilcoxon rank sum test, Pearson chi‐square test and Fisher's exact test were applied. Second, propensity score matching (PSM, ratio 1:10) according to the nearest neighbour was applied to age at admission, CCI, surgical approach (open vs robot‐assisted RP), hospital teaching status, and hospital size between patients with and without paraplegia who underwent RP, to maximally reduce the effect of bias and confounding. Third, multivariable logistic regression models (LRMs) predicting perioperative complications and in‐hospital mortality were fitted after adjustment for clustering at the hospital level using generalized estimation equation (GEE) methodology [13, 15]. Fourth, a sensitivity analysis was conducted using a multivariable GEE model, accounting for clustering at the hospital level within the unmatched population. All analyses and their reporting followed the NIS reporting guideline. Specifically, exact counts and associated proportions were not reported for samples with membership <11 [8]. All tests were two sided, with a significance level set at *P* < 0.05. R software environment was used for statistical computing and graphics (R version 4.2.2; R Foundation for Statistical Computing, Vienna, Austria).

## Results

### Descriptive Characteristics of the Study Population

Within the NIS 2000–2019, we identified 260 302 patients with non‐metastatic PCa who underwent RP between 2000 and 2019. Of those, there were 223 (0.1%) patients with paraplegia (Table 1). Patients with paraplegia were younger (age 60 vs 62 years; *P* = 0.002) and more frequently harboured CCI ≥3 (13% vs 2.2%, *P* < 0.001). Conversely, no statistically significant differences were recorded in surgical approach, teaching hospital status, and hospital size between patients with and without paraplegia undergoing RP.

**Table 1:** Descriptive characteristics of patients with non‐metastatic PCa undergoing RP according to paraplegia status within the NIS from 2000 to 2019 before and after PSM (1:10).

Characteristic	Before PSM (1:10)			After PSM (1:10)^‡^		
	Patients with paraplegia *N* = 223 (0.1%)	Patients without paraplegia *N* = 260 079 (99.9%)	*P* *	Patients with paraplegia *N* = 223 (100%)	Patients without paraplegia *N* = 2230 (0.8%)	*P* *
Age at admission, years, median (IQR)	60 (55–66)	62 (57–67)	**0.002**	60 (55–66)	60 (55–66)	>0.9
CCI, *n* (%)^†^						
0–1	172 (77.0)	241,498 (93)	<**0.001**	172 (77.0)	1721 (77.0)	>0.9
2	21 (9.4)	12 981 (5.0)		21 (9.4)	209 (9.4)	
≥3	30 (13.0)	5600 (2.2)		30 (13.0)	300 (13.0)	
Surgical approach, *n* (%)						
Open	139 (62.0)	151 830 (58.0)	0.2	139 (62.0)	1381 (62.0)	>0.9
Robot assisted	84 (38.0)	108 249 (42.0)		84 (38.0)	849 (38.0)	
Teaching hospital status, *n* (%)	143 (64.0)	71 784 (66.0)	0.5	143 (64.0)	1426 (64.0)	>0.9
Hospital size, *n* (%)						
Large (≥400 beds)	146 (65.0)	172 350 (66.0)	>0.9	146 (65.0)	1,475 (67.0)	>0.9
Medium (200–399 beds)	50 (22.0)	58 696 (23.0)		50 (22.0)	496 (21.0)	
Small (<200 beds)	27 (12.0)	29 033 (11.0)		27 (12.0)	259 (12.0)	

Bold values statistically significant at *P* < 0.05.

* Wilcoxon rank sum test; Pearson's chi‐square test;

^†^ In patients with paraplegia, 2 points were removed to compare the additional comorbidity besides their condition.

^‡^ Matched for age at admission, CCI, surgical approach, teaching hospital status, and hospital size.

### Propensity Score Matching between Patients with and Without Paraplegia who underwent RP

For purpose of pre‐planned comparisons, we relied on 1:10 PSM to maximally reduce differences between patients with and without paraplegia who underwent RP, regarding patient (age at admission and CCI), surgery (open vs robot‐assisted RP), and hospital characteristics (teaching status and size). After PSM, 223 of 223 (100%) patients with paraplegia who underwent RP and 2230 of 260 079 (0.9%) patients without paraplegia who underwent RP that most closely mirrored the characteristics of their counterparts, according to principles of case–control analyses, were included in further considerations [7, 14, 15, 16, 17, 18]. No statistically significant residual differences were recorded for patient (age at admission and CCI), surgery (robot‐assisted vs open approach), and hospital characteristics (teaching hospital status and hospital size; all *P* > 0.9; Table 1).

### Adverse In‐Hospital Outcomes after 1:10 PSM and Multivariable Adjustment

After 1:10 PSM, followed by multivariable logistic regression adjusted for age, CCI, surgical approach, hospital size, and teaching status, paraplegia was independently associated with significantly higher rates of several adverse in‐hospital outcomes. Overall complications occurred more frequently in patients with paraplegia (35% vs 18%, *P* < 0.001; adjusted odds ratio [aOR] 2.4, 95% CI 1.8–3.3). Similar associations were observed for intraoperative complications (13% vs 5%, *P* < 0.001; aOR 2.8, 95% CI 1.8–4.2), cardiac complications (9% vs 3.7%, *P* < 0.001; aOR 2.8, 95% CI 1.8–4.4), pulmonary complications (5.8% vs 2.4%, *P* = 0.002; aOR 2.6, 95% CI 1.3–4.9), and genitourinary complications (4.0% vs 1.5%, *P* = 0.001; aOR 3.5, 95% CI 1.7–6.7). Patients with paraplegia were also more likely to receive blood transfusions (11% vs 6.7%, *P* = 0.01; aOR 1.8, 95% CI 1.2–2.8), and to experience infectious (<4.9% vs <0.5%, *P* = 0.01; aOR 6.2, 95% CI 1.7–22.3) and wound complications (<4.9% vs <0.5%, *P* = 0.02; aOR 8.2, 95% CI 1.8–38.2). Prolonged length of stay (≥75th percentile) was more common (60% vs 34%, *P* < 0.001; aOR 1.7, 95% CI 1.3–2.1), and in‐hospital mortality was also significantly higher (<4.9% vs <0.5%, *P* = 0.003; aOR 10.7, 95% CI 2.8–40.7). There were no significant differences for vascular (*P* = 0.07) or gastrointestinal complications (*P* = 0.4), and paraplegia was not an independent predictor in either case (Table 2).

**Table 2:** Adverse in‐hospital outcomes (overall complications, intraoperative complications, postoperative complications, and mortality) and length of stay in RP patients after 1:10 PSM, stratified according to paraplegia status.

Outcome of interest, *n* (%)	After 1:10 PSM					Before 1:10 PSM	
	Patients with paraplegia, *N* = 223 (100%)*	Patients without paraplegia, *N* = 2230 (0.9%)*	*P* *	Multivariable^†^ RR/OR (95% CI)	*P* ^†^	Multivariable^†^ RR/OR (95% CI)	*P* ^†^
Overall complications	78 (35.0)	406 (18)	**<0.001**	2.4 (1.8–3.3)	**<0.001**	2.4 (1.8–3.1)	**<0.001**
Intraoperative complications	29 (13.0)	111 (5.0)	**<0.001**	2.8 (1.8–4.3)	**<0.001**	3.5 (2.3–5.1)	**<0.001**
**Postoperative complications**							
Infectious complications	<11 (<4.9)	<11 (<0.5)	**0.01**	6.2 (1.7–22.3)	**0.005**	5.2 (1.9–13.8)	**<0.001**
Pulmonary complications	13 (5.8)	53 (2.4)	**0.002**	2.6 (1.4–4.9)	**0.004**	2.8 (1.6–5.0)	**<0.001**
Genitourinary complications	11 (4.9)	33 (1.5)	**0.001**	3.5 (1.7–7.2)	**<0.001**	3.6 (1.8–6.7)	**<0.001**
Wound complications	<11 (<4.9)	<11 (<0.5)	**0.02**	8.2 (1.9–36.8)	**0.006**	4.8 (1.5–15.0)	**0.007**
Cardiac complications	20 (9.0)	82 (3.7)	**<0.001**	2.8 (1.7–4.9)	**<0.001**	2.9 (1.8–4.7)	**<0.001**
Vascular complications	<11 (<4.9)	<11 (<0.5)	0.08	3.4 (0.9–13.5)	0.08	2.5 (0.7–8.2)	0.1
Gastrointestinal complications	16 (7.2)	129 (5.8)	0.4	1.3 (0.7–2.2)	0.4	1.4 (0.9–2.3)	0.1
Blood transfusion	25 (11.0)	150 (6.7)	**0.01**	1.8 (1.2–2.7)	**0.01**	1.6 (1.1–2.5)	**0.01**
Length of stay ≥ 75th percentile^‡^	134 (60)	763 (34)	**<0.001**	1.7 (1.5–1.9)	**<0.001**	1.7 (1.5–1.9)	**<0.001**
In‐hospital mortality	<11 (<4.9)	<11 (<0.5)	**0.003**	10.7 (2.6–44.1)	**<0.001**	18.6 (6.2–55.6)	**<0.001**

Bold values statistically significant at *P* < 0.05. Multivariable LRMs predicting the effect of paraplegia on adverse in‐hospital outcomes. RR, rate ratio.

* Pearson's chi‐square test.

^†^ Multivariable regression models predicting adverse in‐hospital outcomes. Adjustment was made for age at admission, CCI, surgical approach, teaching hospital status, and hospital size.

^‡^ Length of stay ≥75th percentile: ≥3 days.

### Multivariable LRMs Testing the Effect of Paraplegia: A Sensitivity Analysis on Adverse In‐Hospital Outcomes Within the Unmatched Cohort

After multivariable adjustment for age at admission, CCI, surgical approach, teaching hospital status, and hospital size, presence of paraplegia independently predicted higher rates of adverse in‐hospital outcomes in 10 of 12 examined categories. Specifically, presence of paraplegia independently predicted 18.6‐fold higher rate of in‐hospital mortality (aOR 18.6; *P* < 0.001), 5.2‐fold higher rate of infectious complications (aOR 5.2; *P* < 0.001), 4.8‐fold higher rates of wound complications (aOR 4.8; *P* = 0.007), 3.6‐fold higher rate of genitourinary complications (aOR 3.6; *P* < 0.001), 3.5‐fold higher rates of intraoperative complications (aOR 3.5; *P* < 0.001), 2.9‐fold higher rate of cardiac complications (aOR 2.9; *P* < 0.001), 2.8‐fold higher rates of pulmonary complications (aOR 2.8; *P* < 0.001), 2.4‐fold higher rates of overall complications (aOR 2.4; *P* < 0.001), 1.7‐fold higher rates of length of stay ≥75th‐percentile (aOR 1.7; *P* < 0.001) and 1.6‐fold higher rate of blood transfusion (aOR 1.6; *P* = 0.01). Conversely, paraplegia did not affect vascular (*P* = 0.1) and gastrointestinal (*P* = 0.1) complications (Table 2).

## Discussion

The association between paraplegia and adverse in‐hospital outcomes after RP is virtually unknown. Only one previous report based on 14 patients with paraplegia examined that concept [6]. To address this knowledge gap, we relied on a large‐scale population‐based cohort within the NIS (2000–2019). We made several noteworthy observations as follows.

First, we identified 223 (0.1%) patients with paraplegia within 260 302 individuals treated with RP between 2000 and 2019. This number (*n* = 223) significantly exceeds the 14 observations previously described by Gammon et al. (1993–2002) [6]. The current population of patients with paraplegia provides a substantially more robust cohort of individuals of interest. Moreover, it provides a more contemporary comparison opportunity between patients with and without paraplegia who underwent RP then previously reported (2000–2019 vs 1993–2002) [6]. Additionally, unlike Gammon et al. [6], the present analysis was based on standard adverse in‐hospital outcomes’ analysis format that focused on overall complications, perioperative complications, in‐hospital mortality, and length of stay. This specific format was used in several previous analyses examining RP, as well as other surgical procedures [7, 14, 15, 16, 17, 18]. Consequently, this format allows comparisons with these previous studies [7, 14, 15, 16, 17, 18].

Second, we identified important differences in patient characteristics that distinguish patients with and without paraplegia who underwent RP. Specifically, patients with paraplegia who underwent RP were younger (age 60 vs 62 years; *P* = 0.002) and had a higher number of comorbidities (CCI ≥3: 13% vs 2.2%; *P* < 0.001). However, patients with paraplegia who underwent RP were not different regarding the rates of open vs robot‐assisted RP. Similarly, hospital characteristics, defined as teaching hospital status and hospital size, also did not differ between patients with and without paraplegia who underwent RP. Based on important age at admission and CCI differences between patients with and without paraplegia who underwent RP, it is crucial to rely on PSM, as well as on additional multivariable adjustment for residual differences, when adverse in‐hospital outcomes are compared, as was done in the present study, but not in the previous report of Gammon et al. [6].

Third, we relied on PSM to minimise biases and confounders. This consideration is also particularly important, when a small cohort (223 patients with paraplegia who underwent RP) is compared to an exponentially larger cohort (260 079 patients without paraplegia who underwent RP). According to principles of epidemiology, in comparisons between cases (patients with paraplegia who underwent RP) and controls (patients without paraplegia who underwent RP), the characteristics of controls should ideally perfectly match the characteristics of cases, except for the condition of interest, namely paraplegia [7, 14, 15, 16, 17, 18]. To satisfy this condition, for each patient with paraplegia, 10 controls without paraplegia were identified based on PSM criteria (age at admission, CCI, surgical approach, teaching hospital status, and hospital size). After PSM all 223 patients with paraplegia who underwent RP (100%) and 2230 of 260 079 (0.9%) control patients without paraplegia who underwent RP were included in further analyses. Both groups exhibited virtually the same patient (age at admission and CCI), surgical (open vs robot‐assisted approach), and hospital characteristics (teaching hospital status and hospital size; all *P* > 0.9).

Fourth, we tested for differences in adverse in‐hospital outcomes between patients with and without paraplegia who underwent RP within the PSM population. In 10 of 12 categories, patients with paraplegia who underwent RP had significantly higher absolute rates of adverse in‐hospital outcomes. For example, patients with paraplegia exhibited higher in‐hospital mortality (<4.9% vs <0.5%; *P* = 0.003), higher rates of overall complications (35% vs 18%; *P* < 0.001), intraoperative complications (13% vs 5%; *P* < 0.001), blood transfusions (11% vs 6.7%; *P* = 0.01), in addition to longer hospital stay (60% vs 34%; *P* < 0.001). Additionally, despite a low number of observations, patients with paraplegia exhibited higher absolute rates of wound (<4.9% vs <0.5%; *P* = 0.01) and infectious complications (<4.9% vs <0.5%; *P* = 0.02). In multivariable analyses, presence of paraplegia independently predicted 10.7‐fold higher in‐hospital mortality, 2.4‐fold higher rate of overall complications, 2.8‐fold higher rate of intraoperative complications, 1.8‐fold higher rate of blood transfusions, and 1.7‐fold higher rate of length of stay ≥75th percentile.

Despite a low number of observations, paraplegia also independently predicted 8.2‐fold higher rate of wound and 6.2‐fold higher rate of infectious complications. These observations provide robust proof that patients with paraplegia should be considered for RP only after very careful consideration. Specifically, the significantly higher risks of in‐hospital mortality, perioperative complications, and prolonged hospitalisation should be extensively discussed at informed consent prior to treatment decision‐making in patients with paraplegia. Finally, alternative treatment modalities, such as radiotherapy, should also be strongly considered [19].

Fifth, a sensitivity analysis was conducted using a multivariable GEE model, which accounted for clustering at the hospital level and did not include PSM. The results closely mirrored those obtained in the matched cohort and did not change the overall interpretation of the associations observed for each adverse in‐hospital outcome. The largest discrepancy between the two approaches was noted for in‐hospital mortality, with an aOR of 10.7 in the PSM analysis vs 18.6 in the non‐PSM model. Nonetheless, the clinical interpretation remained consistent. Similar stability in effect estimates was observed across all other adverse outcomes, reinforcing the robustness and reliability of our findings.

Sixth, the observations made within the present study regarding the effect of paraplegia on adverse in‐hospital outcomes may only be compared with a single study reported by Gammon et al. [6]. In that study only 14 patients with paraplegia were described with respect to specific adverse in‐hospital outcomes that consisted of anaemia, wound infections, UTIs, atelectasis, and postoperative ileus. However, Gammon et al. [6] neither relied on PSM nor applied multivariable analyses. Finally, Gammon et al. [6] did not rely on standard metrics for identifying and quantifying the severity of adverse in‐hospital outcomes that were used in the present studies as well as in multiple previous analyses of the same endpoints. These differences preclude any attempt at a valid comparison.

Seventh, previous studies examining the association between presence of paraplegia and adverse in‐hospital outcomes in other surgical procedures than RP are also scarce. The largest of those examined the effect of paraplegia on adverse in‐hospital outcomes in a cohort that included 185 patients with paraplegia treated with radical cystectomy for bladder cancer [7]. In that study, paraplegia exerted an equally important effects on adverse in‐hospital outcomes (in‐hospital mortality and intraoperative complications), as recorded in the present report. Moreover, former objective studies quantifying the effect of paraplegia on adverse in‐hospital outcomes on other major cancer surgeries are not available. However, Benjamin et al. [20] reported higher rates of postoperative complications in 274 patients with paraplegia undergoing emergency abdominal surgeries, namely appendectomy, cholecystectomy, or other unspecified hepato‐pancreato‐biliary procedures, when compared to control patients without paraplegia. Similarly, in addition to our results, these studies may suggest the need for heightened perioperative monitoring and tailored surgical strategies in this population.

Taken together, this study identified significant differences between patients with and without paraplegia who underwent RP. In particular, patients with paraplegia who underwent RP were younger and had higher rates of comorbidities. Due to these differences, both PSM and multivariable adjustment were used to minimise bias and confounders. Specifically, after PSM for patient, surgical, and hospital characteristics, paraplegia independently predicted higher rates of adverse in‐hospital outcomes. These consisted of 10.7‐fold higher in‐hospital mortality, 2.4‐fold higher rate of overall complications, 2.8‐fold higher rate of intraoperative complications, 1.8‐fold higher rate of blood transfusions, and 1.7‐fold higher rate of length of stay ≥75th percentile, in addition to 8.2‐fold higher rate of wound complications and 6.2‐fold higher rate of infectious complications. These observations convincingly indicate that RP consideration should be made with utmost caution in patients with paraplegia. Specifically, the significantly elevated risks of in‐hospital mortality, perioperative complications, and prolonged hospitalisation support the need for comprehensive preoperative risk assessment, detailed informed consent discussions, and consideration of non‐surgical treatment alternatives. Despite the novelty of our observations, the present study is not devoid of limitations.

First, selection and reporting biases may have remained due to the retrospective nature of the NIS. This limitation applies to the present study as well as to all previous analyses relying on the NIS [13, 14, 16, 21] or other large‐scale retrospective databases, such as the Surveillance Epidemiology and End Results database. Second, despite the very large size of the NIS, patients with paraplegia were rare and, based on NIS reporting limitations, specific details could not be provided for some of the comparisons when patients count were <11. Instead, only relative metrics, such as OR, could be provided. Additionally, the number of details included in the present analysis was also limited due to the nature of the NIS. For example, the specific nature of paraplegia and its duration, as well as the level of spinal cord injury were not available. Additionally, despite the use of a standardised approach, a limited number of details was available regarding the nature of the complications that were examined. For example, the amount of blood units used in transfusion, as well as Clavien–Dindo classification of surgical complications were not known. Specifically, all of the complications examined within the current database were recorded retrospectively. Last but not least, the NIS exclusively provides in‐hospital data. Therefore, it was not possible to assess further complications after the patient was discharged after RP. Nevertheless, the present study provides the most detailed and methodologically structured analysis on the association between paraplegia and adverse in‐hospital outcomes in patients with paraplegia who underwent RP.

## Conclusion

Although patients with paraplegia who undergo RP patients are rare, adverse in‐hospital outcomes are substantially more frequent in such individuals. These observations should be carefully considered in clinical decision making and informed consent prior to RP if such a procedure is contemplated in patients with paraplegia.

## Disclosure of Interests

The authors declare no conflicts of interest related to this manuscript. All authors have completed the International Committee of Medical Journal Editors (ICMJE) disclosure form and have disclosed any relationships or activities that could be perceived to influence the submitted work. No financial or non‐financial interests relevant to the content of this article were reported.

## Supporting information


**Table S1.** The ICD‐9‐CM and ICD‐10 codes for non‐metastatic PCa, paraplegia and adverse in‐hospital outcomes.
