# Supplementary material for: Adverse in‐hospital outcomes in patients with paraplegia who undergo radical prostatectomy
Source: BJU Int. 2025 Oct 10;137(1):209–15. doi: 10.1111/bju.70021 (PMC12690342; doi:10.1111/bju.70021)
Supplement: Supplementary file 1 — Table S1. The ICD‐9‐CM and ICD‐10 codes for non‐metastatic PCa, paraplegia and adverse in‐hospital outcomes. [file BJU-137-209-s001.docx]

**Supplementary Table 1.** ICD-9-CM and ICD-10 codes for non-metastatic prostate cancer, paraplegia and adverse in-hospital outcomes

| **Characteristics** | **ICD-9-CM codes** | **ICD-10 codes** |
| --- | --- | --- |
| **Paraplegia** | 344.1: Paraplegia  342.x: Hemiplegia and hemiparesis  334.1: Hereditary spastic paraplegia  343.x: Infantile cerebral palsy  344.0–344.6: Other paralytic syndromes  344.9: paralysis, unspecified | G04.1: Tropical spastic paraplegia  G11.4: Hereditary spastic paraplegia  G80.1: Spastic diplegic cerebral palsy  G80.2: Spastic hemiplegic cerebral palsy  G81.x: Hemiplegia and hemiparesis  G82.x: Paraplegia (paraparesis) and quadriplegia (quadriparesis)  G83.0–G83.4: Other paralytic syndromes  G83.9: Paralytic syndrome, unspecified |
| **Metastatic prostate cancer patients (exluded from the analyses)** | 1970,1971,1972,1973,1974,1975,1976,  1977,1978,1980,1981,1982,1983,1984,  1985,1986,1987,1988,19881,19882,  19889,1960,1961,1962,1963,1965,1966,  1968,1969 | C78, C780, C7800, C7801, C7802, C781,  C782, C783, C7830, C7839, C784, C785,  C786, C787, C788, C7880, C7889, C79,  C790, C7900, C7901, C7902, C791, C7910, C7911, C7919, C792, C793, C7931, C7932, C794, C7940, C7949, C795, C7951, C7952, C796, C7960, C7961, C7962, C797, C7970, C7971, C7972, C798, C7981, C7982, C7989, C799, C77, C770, C771, C772, C773, C774, C775, C778, C779 |
| **Intraoperative**  **complcations** | 997.02, 998.11, 998.12, 997.1, 998.2, 997.5, 997.49, 349.31, 997.99, 997.01,  997.09, 998.89, 997.39, 995.86, V58.89, 909.3, 999.9, 668.90, 995.22, 995.4, 995.89, 995.24, 995.0, 909.5, 998.11, 998.12 | G97.3xx, N99.61, N99.62, N99.71, N99.72, N99.81,K91.61, K91.62, K91.71, K91.72, K91.81, L76.01, L76.02, I97.410, I97.411, I97.418, I97.42, I97.51, I97.52, I97.711, I97.791, I97.810, I97.811, I97.88, G97.41, G97.48, G97.49, G97.81, M96.810, M96.811, M96.820, M96.821, M96.89,  E36.01, E36.02, E36.11, E36.12, E36.8,  J95.61, J95.62, J95.71, J95.72, J95.88,  T883.xx, T884.xx, T885.xx, T886.xx, T887.xx, T888.xx, T889.xx, D78.0.xx, D78.1.xx, D78.81, M96.811 |
| **Infectious**  **complications** | 53641, 51901, 9985, 993, 038, 0545, 7907, 99591, 99592 | T8140.xx, T8144.xx, A40.xx, A41.xx, B007, R7881, R65.xx, A493, B960, B961, B966, B967, B9689, B9681,V5889, |
| **Pulmonary**  **complications** | 5180, 5184, 514, 4660, 46611, 46619, 4800, 4801, 4802, 4803, 4808, 4809, 481,  4820, 4821, 4822, 4823, 4824, 4828, 4829, 5070, 51881, 4830, 4831, 4838, 485, 486, 41881, 51882, 7991,9973 | J09.xx, J10.xx, J11.xx, J12.xx, J13.xx, J14.xx, J15.xx, J16.xx, J17.xx, J18.xx, J20.xx, J21.xx, J80.xx, J81.xx,J90, J91.xx, J93.xx, J9600, J9601, J9602, J9620, J9621, J9622, J969.xx, R092, J9581.xx, J9582.xx, J9585.xx, J9586.xx, J9589 |
| **Genitourinary**  **complications** | 59010, 59011, 5902, 59080, 59081, 5909, 591, 5933, 5934, 5935, 59381,  59382, 59589, 5961, 5962, 5966, 9975 | N10, N136, N130, N131, N132, N133, N280, N2884, N2885, N45.xx, N49.xx, N34.xx, N390, N151, N91.xx, N992.xx, N993.xx, N994.xx, N995.xx, N9989 |
| **Wound**  **complications** | 9983, 99830, 99831, 99832, 99833, 9985, 99859, 99851, 9986, 567 | T81.3.xx, T8141.xx, T8142.xx, T8143.xx, |
| **Cardiac**  **complications** | 428.1, 428.20, 428.21, 428.23, 428.30, 428.31, 428.33, 428.40, 428.41, 428.43, 428.9, 4100, 4101, 4102, 4103, 4104, 4105, 4106, 4107, 4108, 4109, 4110, 40211, 40291, 4275, 9971 | I50.1, I50.21, I50.23, I50.31, I50.33, I50.40, I50.41, I50.43, I50.811, I50.81, I50.814, I50.82, I50.83, I50.84, I50.89, I50.9, I160, I161, I169, I20.xx, I21.xx, I22.xx, I23.xx, I24.xx, I44.xx, I48.x, I46.2, I46.8, I46.9, I47.xx, I51.xx, I971.xx, I9761.xx, I9782, I9789 |
| **Vascular**  **complications** | 4151, 41511, 41512, 41519, 4512, 45181, 4519, 4538, 4539, 9972, 9992, 44422, 44481, 433, 4330, 4331, 4332, 4333, 4338, 4339, 434, 4340, 4341, 4349, 436, 437, 4371, 4372, 4374, 4373, 4375, 4376, 4377, 4378, 4379 | I81, I82.210, I82.220, I82.23, I82.290, I82.4.xx, I82.6.xx, I82.A1.xx, I82.B.1.xx, I82.C1.xx, I82.8.xx, I82.890, I82.90, I80.xx, I71.xx, I77.7xx, I74.xx, I75.xx, I76, I26.xx, I28.8, I28.9 |
| **Gastrointestinal**  **complications** | 5310, 5311, 5312, 5313, 5320, 5321, 5322, 5323, 5400, 5401, 5409, 5600, 5601, 5602, 5603, 5608, 5609, 7876, 9974, 5692, 5693, 5695, 5696, 5793, 00845 | K250, K251, K253, K259, K260, K261, K263, K269, K270, K271, K273, K279, K280, K281, K283, K289, K35.xx, K36.xx, K45.xx, K65.xx, K66.xx, K67.xx, K68.xx, K610, K611, K626, K630, K631, K632, K633, K6181.xx, K9182, K9183, K9189 |
| **Blood transfusions** | 9902, 9903, 9907, 9904, 9905, 9906 | 30233H0, 30233H1, 30233K0, 30233K1, 30233L0, 30233L1,30233N0, 30233N1, 30233P0, 30233P1, 30233R0, 30233R1, 30233T0, 30233T1 |
| **Critical care therapy** | IMV: 9670, 9671, 9672  PEG: 0DH63UZ, 0DH64UZ  Dialysis for AKI: 3995  TPN: 9915  TRA: 311, 3121, 3129 | IMV: 5A1935Z, 5A1945Z, 5A1955Z  PEG: 4311  Dialysis for aki: 5A1D70Z, 5A1D80Z, 5A1D90Z, 5A1D00Z, 5A1D60Z  TPN: 3E0336Z, 3E0436Z, 3E0536Z, 3E0636Z  TRA: 0B110F4, 0B110Z4, 0B113F4, 0B113Z4, 0B114F4, 0B114Z4 |
